# Characterizing the transition from immune response to tissue repair after myocardial infarction by multiparametric imaging

**DOI:** 10.1007/s00395-022-00922-x

**Published:** 2022-03-11

**Authors:** Annika Hess, Tobias Borchert, Tobias L. Ross, Frank M. Bengel, James T. Thackeray

**Affiliations:** 1 Department of Nuclear Medicine, Hannover Medical School, Carl-Neuberg-Str. 1, 30625 Hannover, Germany; 2 Present Address: Cardior Pharmaceuticals GmbH, Hannover, Germany

**Keywords:** Cardiac repair, Inflammation, Positron emission tomography

## Abstract

Persistent inflammation following myocardial infarction (MI) precipitates adverse outcome including acute ventricular rupture and chronic heart failure. Molecular imaging allows longitudinal assessment of immune cell activity in the infarct territory and predicts severity of remodeling. We utilized a multiparametric imaging platform to assess the immune response and cardiac healing following MI in mice. Suppression of circulating macrophages prior to MI paradoxically resulted in higher total leukocyte content in the heart, demonstrated by increased CXC motif chemokine receptor 4 (CXCR4) positron emission tomography imaging. This supported the formation of a thrombus overlying the injured region, as identified by magnetic resonance imaging. The injured and thrombotic region in macrophage depeleted mice subsequently showed active calcification, as evidenced by accumulation of ^18^F-fluoride and by cardiac computed tomography. Importantly, macrophage suppression triggered a prolonged inflammatory response confirmed by post-mortem tissue analysis that was associated with higher mortality from ventricular rupture early after occlusion and with increased infarct size and worse chronic contractile function at 6 weeks after reperfusion. These findings establish a molecular imaging toolbox for monitoring the interplay between adverse immune response and tissue repair after MI. This may serve as a foundation for development and monitoring of novel targeted therapies that may include immune modulation and endogenous healing support.

**Supplementary Information:**

The online version contains supplementary material available at 10.1007/s00395-022-00922-x.

## Introduction

Local tissue inflammation after acute myocardial infarction (MI) plays a crucial role in cardiac repair. Balance between inflammatory and reparative leukocyte subtypes is required for optimal repair, leading to preserved cardiac function and a stable collagen-rich scar of limited size [9, 18]. Effective repair is mediated by an orchestrated assortment of leukocyte subtypes to transition from pro-inflammatory to reparative signaling [7, 9, 13]. Complete suppression of inflammation leads to more severe remodeling [16]. Selective inhibition of some leukocyte populations may be beneficial [13], but complete suppression of macrophages prior to experimental MI can evoke higher mortality and impede repair [1, 10, 29].

Molecular imaging facilitates non-invasive monitoring of the immune response and repair processes after MI. The intensity of the inflammation signal predicts the severity of ventricle remodeling after acute MI [13, 22]. Moreover, single time inhibition of CXC motif chemokine receptor 4 (CXCR4) to reduce inflammatory cell infiltration improves outcome after experimental MI, characterized by a shift in proportions of left ventricle inflammatory leukocytes toward reparative subtypes [13, 30]. Nonetheless, molecular imaging of inflammation indicates maintained or even increased signal after therapy [13]. Indeed, different molecular imaging radiotracers exhibit accumulation in distinct leukocyte subtypes [2].

We hypothesized that multi-tracer molecular imaging would provide insights into the leukocyte subtype content in the infarct territory early after ischemic damage. To this end, we depleted monocyte-derived macrophage content using clodronate-loaded liposomes prior to MI and assessed inflammation and cardiac morphology using multiparametric non-invasive molecular imaging and terminal histopathology. This approach provides a molecular imaging arsenal to monitor the interplay between adverse immune response and tissue repair.

## Methods

### Animals

All animal experiments were approved by the local state authority (Landesamt für Verbraucherschutz und Lebensmittelsicherheit) and conducted in accordance with European and international guidelines. Male C57BL6/N mice (10 weeks old) were purchased from Charles River, Germany and housed in groups under a 14h/10h light/dark cycle with nesting material, standard laboratory diet and water freely available.

### Myocardial infarction models

Mice received either clodronate-loaded liposomes (*n*=72) or control PBS-loaded liposomes (*n*=40) by intravenous injection. After 24 h, mice underwent permanent occlusion (*n*=83), or ischemia/reperfusion (60 min, *n*=18) of the left coronary artery, or sham surgery (*n*=11) as previously described [13]. Briefly, mice received the analgesic butorphanol (2 mg/kg BW), were anaesthetized with isoflurane (4%, 0.8 L/min O2), intubated, and mechanically ventilated (2% isoflurane, 0.8 L/min O2). The thorax was entered through the fourth intercostal space, and the pericardium opened. The left anterior descending coronary artery was identified and ligated around 1 mm below the left auricle either permanently, or transiently for 60 min around a piece of PE10 tubing. Then, the chest wall was closed, anaesthesia stopped, and mice were recovered. Details on the assignment of animals to different experimental groups is summarized in Supplementary Table 1.

### Positron emission tomography (PET) tracer synthesis

The CXCR4 ligand ^68^Ga-pentixafor, which targets a broad range of leukocytes [2], was synthesized using an automated module and CPCR4.2 provided by Scintomics (Fürstenfeldbruck, Germany), as previously described [6, 11]. The 18kD-translocator protein (TSPO) ligand ^18^F-GE180, which preferentially targets pro-inflammatory macrophages [2], was synthesized semi-automatically using a radiochemistry module, as described previously [27], with high radiochemical purity, yield, and specific activity (450–600 GBq/µmol). No-carrier-added ^18^F-fluoride was produced by the ^18^O(p,n)^18^F nuclear reaction with a Siemens ECLYPSE HP cyclotron. The target solution (^18^F-fluoride in ^18^O-water) was transferred via a capillary line directly connected to a self-designed radiosynthesis apparatus in a shielded hot cell. In a custom-made single-use cassette system, the ^18^F-fluoride was captured on an anion exchange cartridge (Sep-Pak Plus QMA light, Waters), washed with water and dried by argon gas. The final product ^18^F-NaF was then eluted and reformulated under 0.9% sodium chloride solution for injection.

### PET imaging

The longitudinal imaging study design is summarized in Supplementary Fig. 1. Mice underwent whole body PET scans using a dedicated small animal camera as described [3, 13]. Cardiac inflammation was assessed using ^68^Ga-pentixafor (12.3±1.1 MBq) 1 day, 3 days and 7 days after surgery (*n*=55). Tracer was injected into a lateral tail vein as a 0.1–0.15 mL bolus under isoflurane anaesthesia. After 50 min conscious uptake, a 10 min static PET scan was acquired. To facilitate signal localization, 19.1±2.8 MBq ^18^F-FDG was injected i.p. subsequently and after 20 min uptake under isoflurane, a second 10 min static PET scan was performed. Instead of ^68^Ga-pentixafor PET, a subgroup of permanent occlusion MI mice (*n*=29) underwent TSPO PET imaging using ^18^F-GE180 (13.6±2.0 MBq) 3 and 7 days after surgery for imaging of cardiac macrophage infiltration. Here, acquisition of 60 min dynamic scans was started simultaneously to tracer injection. At 4 weeks post-surgery, mice underwent a 60 min dynamic PET scan using ^18^F-NaF (18.1±1.2 MBq) to assess microcalcification. Dynamic scans were histogrammed to 32 frames of 5×2, 4×5, 3×10, 8×30, 5×60, 4×300, and 3×600 s. PET images were reconstructed to a 128×128×159 image matrix (0.79 mm pixel size) using a 3D ordered subset expectation maximization/maximum a posteriori (OSEM3D/MAP) algorithm (*β*=0.01, OSEM iterations=2, MAP iterations=18) as described [3, 13]. PET images were analysed semiquantitavely using Inveon Research Workplace 4.2 (Siemens). Cardiac volumes of interest were drawn to derive raw voxel intensity (Bq/cm^3^), and percent injected dose per gram of tissue (%ID/g) was subsequently calculated.

### Computed tomography (CT) imaging

Following every PET scan, a short low dose CT was acquired on a Siemens Inveon small animal camera (Siemens AG, Medical Solutions, Erlangen, Germany) over 360 projections with an exposure time of 100 ms (at 80 kV, 500 µA), and a total acquisition time of 430 s. Images were rebinned to 4×4 with a pixel size of 95.43 µm, a transaxial field of view of 70.24 mm, and an axial field of view of 94.67 mm. Images were reconstructed using a Feldkamp algorithm, and Shepp-Logan filter was applied.

### Single photon emission computed tomography (SPECT) imaging

For infarct size calculation, mice underwent ^99m^Tc-sestamibi perfusion SPECT scans at 6 weeks after surgery using the eXplore speCZT small animal camera (Trifoil Imaging, Chatsworth, CA, USA) equipped with a mouse 7-pinhole collimator and a full ring of cadmium-zinc-telluride (CZT) detectors as described previously [14]. Briefly, mice were anaesthetized using isoflurane, and ^99m^Tc-sestamibi (114.6±14.9 MBq) was injected via lateral tail vein as a 0.10–0.15 mL bolus. After 30 min uptake under isoflurane, the SPECT scan was started over 108 views (30 s/view). Polar map analysis was performed in Munich Heart software [24] for infarct size calculation (% of left ventricle (LV)), with defect defined as territories exhibiting <60% of the normalized maximum activity [14].

### Cardiac magnetic resonance (CMR) imaging

For cardiac functional analysis, mice underwent CMR imaging at 1 week and 6 weeks after surgery on a 7T PharmaScan 70/16 (Bruker BioSpin GmbH, Erlangen, Germany) with self-gated cine acquisition, as described previously [13]. A 72 mm diameter volume transmit coil was used in combination with an anatomically shaped four-element mouse cardiac phased array surface receive coil (Bruker BioSpin). The anaesthetized mouse was positioned in the camera with the heart in the centre field of view. Three localizers (coronal, axial, vertical) were acquired to plan a cardiac horizontal long axis and a cardiac short axis scan with nine 0.9 mm slices covering apex to base. A navigator-based self-gated cine CMR sequence (IntraGate^©^ FLASH, Bruker BioSpin) was used [32] with an echo time of 1.842 ms and a repetition time of 85 ms. Images were reconstructed to 10 frames for each cardiac cycle. Image analysis was performed using Segment v2.0 (Medviso) [12] to derive end systolic (ES) and end diastolic (ED) volumes. LV ejection fraction (EF) was calculated as follows: LVEF = ((LVV_ED_–LVV_ES_)/LVV_ED_) × 100 where LVV is left ventricular volume at ED or ES.

### Correlative measurements

Immunohistochemistry was used to identify myocardial cell infiltration following cardiac injury. Therefore, mice (*n*=28) were sacrificed 1 day, 3 days, and 7 days after MI or sham, and hearts were removed, embedded in TissueTek, and snap frozen in liquid nitrogen. CD68 (clone MCA1957B, Bio-Rad, München, Germany) identified monocytes/macrophages, Ly6G (clone 1A8, Biolegend, San Diego, CA, USA) identified granulocytes, and CD41 (clone MWReg30, Biolegend, San Diego, CA, USA) identified activated platelets in 6 µm cryosections within the myocardium. To estimate the number of infiltrative cells to the infarct area, immunohistochemistry images were quantified using ImageJ software. Positive cells were manually counted using 2–3 views per heart section and 2–5 biological replicates. The average number of positive cells was used for statistical analysis. Masson Trichrome staining (Sigma-Aldrich, Darmstadt, Germany) was employed to analyse scar formation and general morphology. *Ex vivo* autoradiography was performed in 10 µm cryo-slices of myocardial tissue from a subgroup of mice (*n*=9) 3 days after MI to validate *in vivo* CXCR4 imaging signal. Here, ^68^Ga-pentixafor was injected as described above, mice were sacrificed 60 min later, hearts removed, frozen, sliced, and exposed to a phosphor imaging screen (PerkinElmer, MA, USA). Autoradiography images were colocalized with immunohistochemical findings as described [26]. In a subgroup of mice from the longitudinal imaging study (*n*=10), we performed Alizarin red staining in 6 µm cryosections to assess calcification. Overview histological images were acquired using a ZEISS Stemi 508 stereo microscope with a 10–50-fold magnification and ZEISS ZEN Blue software. Background in Masson trichrome overview images was removed using ImageJ software. Detailed images of all histological sections were acquired using a Nikon ECLIPSE Ci Microscope with 200-fold magnification and NIS-Elements BR software.

### Statistics

Data are presented as mean ± standard deviation. Two groups were compared using Student’s *t* test with Welch’s correction where appropriate. Where necessary, Sidak-Bonferroni method was used to correct for multiple comparisons. Three groups were compared using one-way analysis of variance (ANOVA) with Bonferroni’s post-hoc test. Log-Rank test was used to compare survival curves of two groups. Statistical analysis was performed with GraphPad Prism (La Jolla, CA, USA, Version 6.01).

## Results

### Clodronate lowers infiltrating macrophage content in the LV and increases incidence of acute LV rupture

**Fig. 1:**
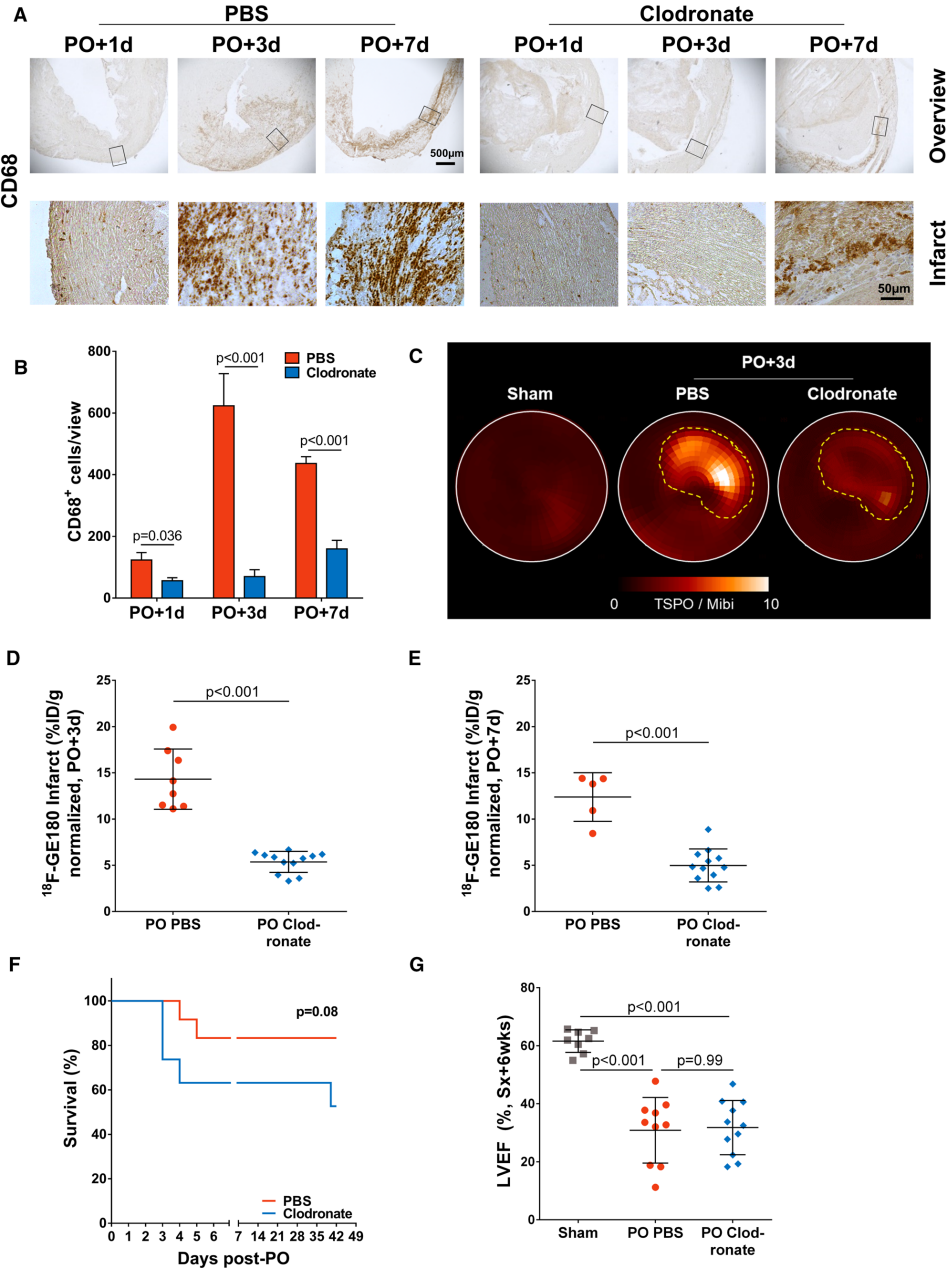
Macrophage depletion successfully reduces macrophage accumulation within the infarcted myocardium. **A** Immunohistochemistry for CD68+ macrophages, and **B** matching quantification demonstrate that macrophage content in the infarct is significantly reduced by macrophage depletion on permanent occlusion MI+1 day, 3 days, and 7 days compared to PBS (*n*=2–5 per timepoint and treatment). Matching the repopulation of blood monocytes after single dose of clodronate-liposomes, there are some macrophages infiltrating the infarct at permanent occlusion MI+7 days. **C** Representative parametric ^18^F-GE180 LV polar maps at 3 days after permanent occlusion normalized to perfusion measured by ^99m^Tc-sestamibi demonstrate reduced infarct TSPO expression in macrophage depleted mice. TSPO expression is significantly reduced in the infarct of macrophage depleted mice at **D** 3 days, and **E** 7 days after permanent occlusion. **F** Macrophage depletion increases incidence of LV rupture within the first 7 days after permanent occlusion 2.2-fold compared to PBS-treated infarct mice, and increases risk of death by chronic heart failure at 6 weeks. **G** In the surviving mice, there is no difference in late LVEF between treatment groups. Statistics: Student’s unpaired t-test with Welch’s correction, or Sidak-Bonferroni method for multiple comparisons where appropriate, one-way ANOVA with Bonferroni’s post-hoc test. *LV* left ventricle, *LVEF* left ventricular ejection fraction, *Mibi* ^99m^Tc-sestamibi, *PO* permanent occlusion, *d* day, *wks* weeks, *Sx* surgery, *TSPO* translocator protein

Pretreatment with clodronate liposomes significantly lowered macrophage content in the infarct and border zone territories at 1 day, 3 days and 7 days after permanent occlusion of the left coronary artery when compared to the PBS control group on histologic analysis, but partial repopulation was observed at 7 days (Fig. 1A, B). Noninvasive *in vivo* imaging using the macrophage-specific TSPO radioligand ^18^F-GE180 confirmed effective depletion. Irrespective of perfusion defect size, uptake of ^18^F-GE180 was lower in the infarct and border zone territory in macrophage depleted mice compared to PBS-treated animals (Fig. 1C–E). Interestingly, macrophage depletion resulted in a marked increase in the incidence of acute LV rupture (2.2-fold, 37 vs 17%)) within the first week after permanent occlusion MI (Fig. 1F), indicating insufficient clearance of debris and impaired scar formation. Further, two macrophage depleted mice after permanent occlusion MI died of chronic heart failure in week 6 (Fig. 1F). All surviving animals showed a significant reduction of contractile function at 6 weeks after permanent occlusion MI when compared to sham-operated animals (Fig. 1G).

### Macrophage depletion leads to sustained neutrophil accumulation in the infarcted myocardium within the first week after MI

**Fig. 2:**
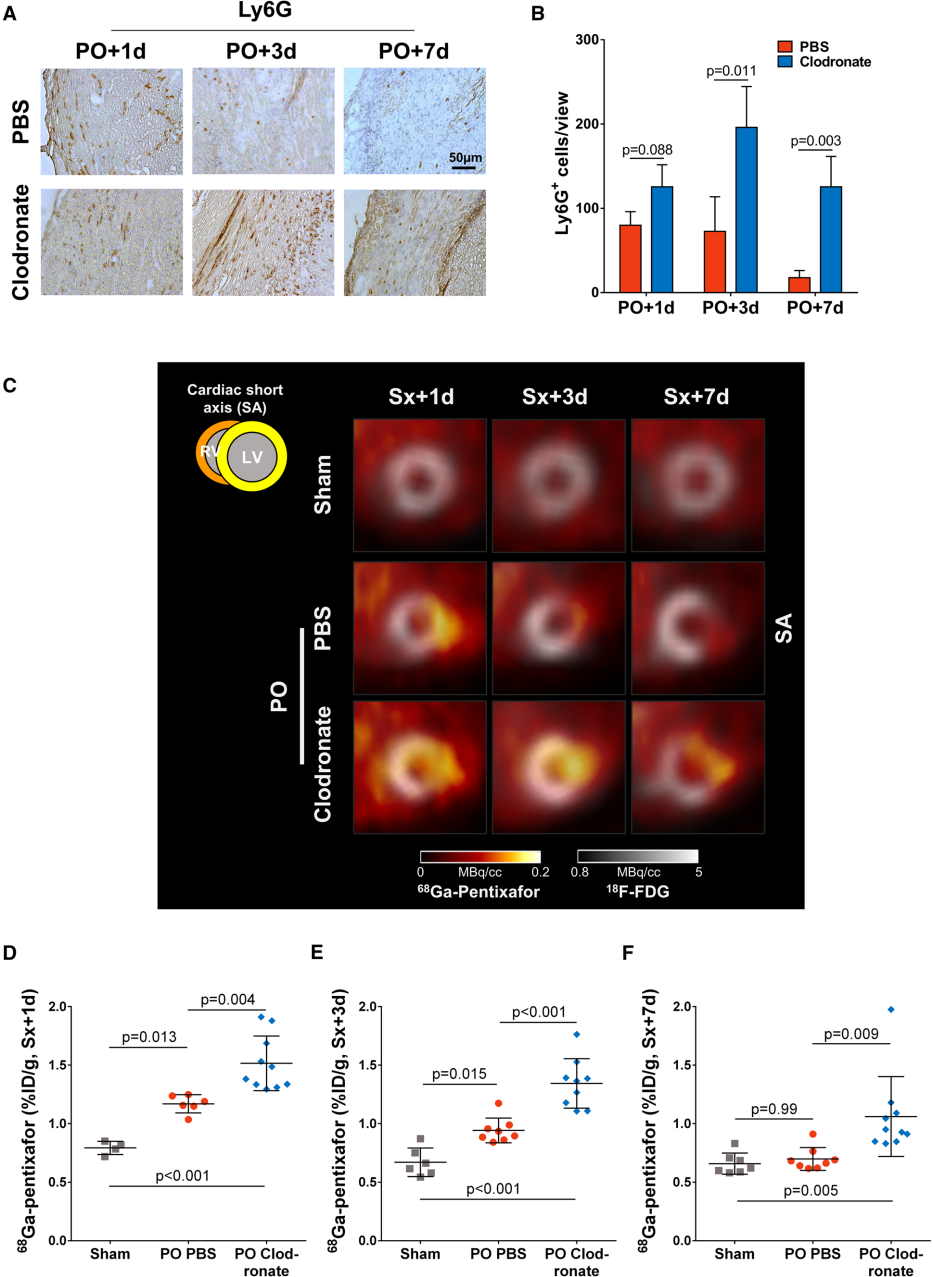
Macrophage depletion increases accumulation of granulocytes, and CXCR4 expression within the infarct. **A** Immunohistochemistry for Ly6G+ granulocytes, and **B** matching quantification show a significant increase in granulocyte accumulation within the infarct territory of macrophage depleted mice at all timepoints (*n*=2–5 per timepoint and treatment). **C** Representative short axis cardiac images using ^68^Ga-pentixafor (colorscale) show increased CXCR4 expression in the ^18^F-FDG defined infarct (greyscale) at permanent occlusion MI+1 day, and 3 days in PBS-treated infarct mice compared to sham. Of note, infarct CXCR4 expression is further increased after macrophage depletion, consistent with presence of an increased number of granulocytes. Semi-quantitative analysis shows that CXCR4 expression in the infarct is significantly higher in macrophage depleted mice compared to PBS-treated mice at **D** permanent occlusion MI+1 day, **E** 3 days, and **F** 7 days. Statistics: Student’s unpaired *t* test with Sidak-Bonferroni method for multiple comparisons, one-way ANOVA with Bonferroni’s post-hoc test. *PO* permanent occlusion, *SA* short axis, *Sx* surgery, *d* day

Histological staining for Ly6G demonstrated a significantly increased granulocyte accumulation in the infarcted myocardial wall over 7 days after permanent occlusion MI in macrophage depleted mice when compared to the PBS control group (Fig. 2A, B). PET imaging using ^68^Ga-pentixafor was performed to investigate infarct CXCR4 expression. As previously demonstrated [13], CXCR4 expression in the infarct territory of PBS-treated mice after permanent occlusion MI was elevated at 1 day and 3 days compared to sham-operated animals, declining by 7 days (Fig. 2C). Matching the increase in granulocyte accumulation within the myocardium, CXCR4 expression in macrophage depleted mice after permanent occlusion MI was significantly elevated compared to PBS-treated mice, and unlike PBS-treated permanent occlusion mice remained elevated compared to sham at 7 days after injury (Fig. 2D–F), despite reduced macrophage infiltration to the damaged region.

### Myocardial injury is associated with intraventricular thrombus formation after macrophage depletion

**Fig. 3:**
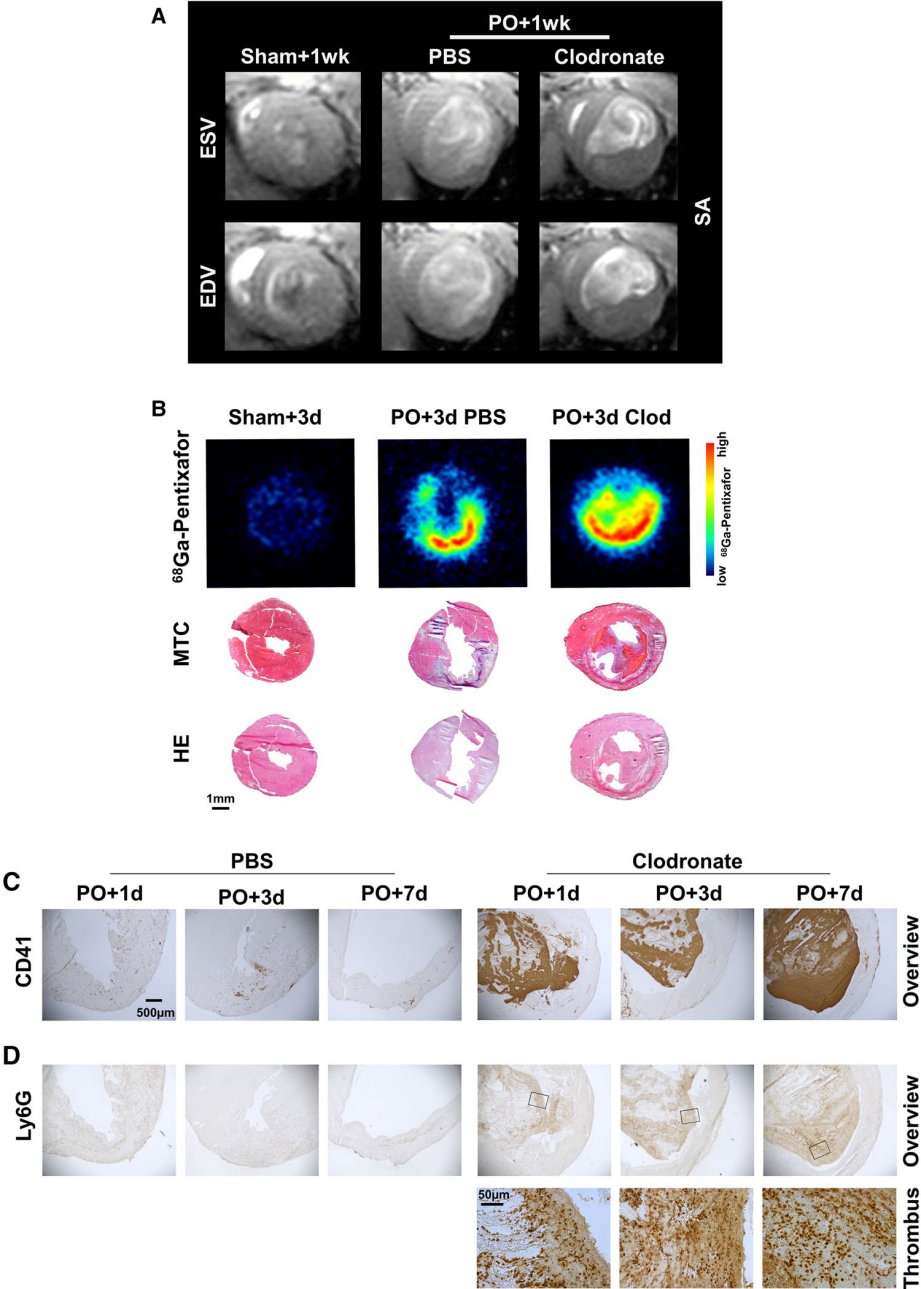
LV thrombus formation after macrophage depletion. **A** Cardiac short axis magnetic resonance images reveal LV thrombus formation at permanent occlusion MI+1 week in macrophage depleted mice. **B** *Ex vivo* autoradiography using ^68^Ga-pentixafor at permanent occlusion MI+3 days demonstrates that increased CXCR4 expression after macrophage depletion is localized not only to the infarct wall, but also to LV thrombus in Masson trichrome and Hematoxylin/Eosin histology. **C, D** Immunohistochemistry at permanent occlusion MI+1 day, 3 days, and 7 days shows that the LV thrombus after macrophage depletion is present from permanent occlusion MI+1 day, and consists of large numbers of CD41+ activated platelets, and Ly6G+ granulocytes. *Clod*, Clodronate, *EDV* end diastolic volume, *ESV* end systolic volume, *HE* Hematoxylin/Eosin, *MTC* Masson trichrome, *PO* permanent occlusion, *SA* short axis, *d* day, *wk* week

CMR imaging at 1 week after surgery demonstrated an intraventricular thrombus affixed to the infarcted myocardium in all macrophage depleted mice after permanent occlusion MI, which was absent in macrophage depleted sham animals or PBS-treated permanent occlusion mice (Fig. 3A, Supplementary Video 1). *Ex vivo* autoradiography showed an increase in CXCR4 expression colocalized not only to infarcted myocardium, but also to the thrombotic region as defined by histology (Fig. 3B). Immunostaining for CD41 and Ly6G (Fig. 3C, D) showed consistent accumulation of granulocytes in the area of platelet-rich thrombus. Taken together, these results suggest that macrophage depletion triggers an endocardial thrombus, probably through impaired endothelial integrity, which contributes to further granulocyte accumulation and aggravation of inflammation, and underlies the persistently elevated *in vivo* infarct CXCR4 PET signal.

### Tissue damage and thrombus formation after macrophage depletion trigger active calcification post MI

**Fig. 4:**
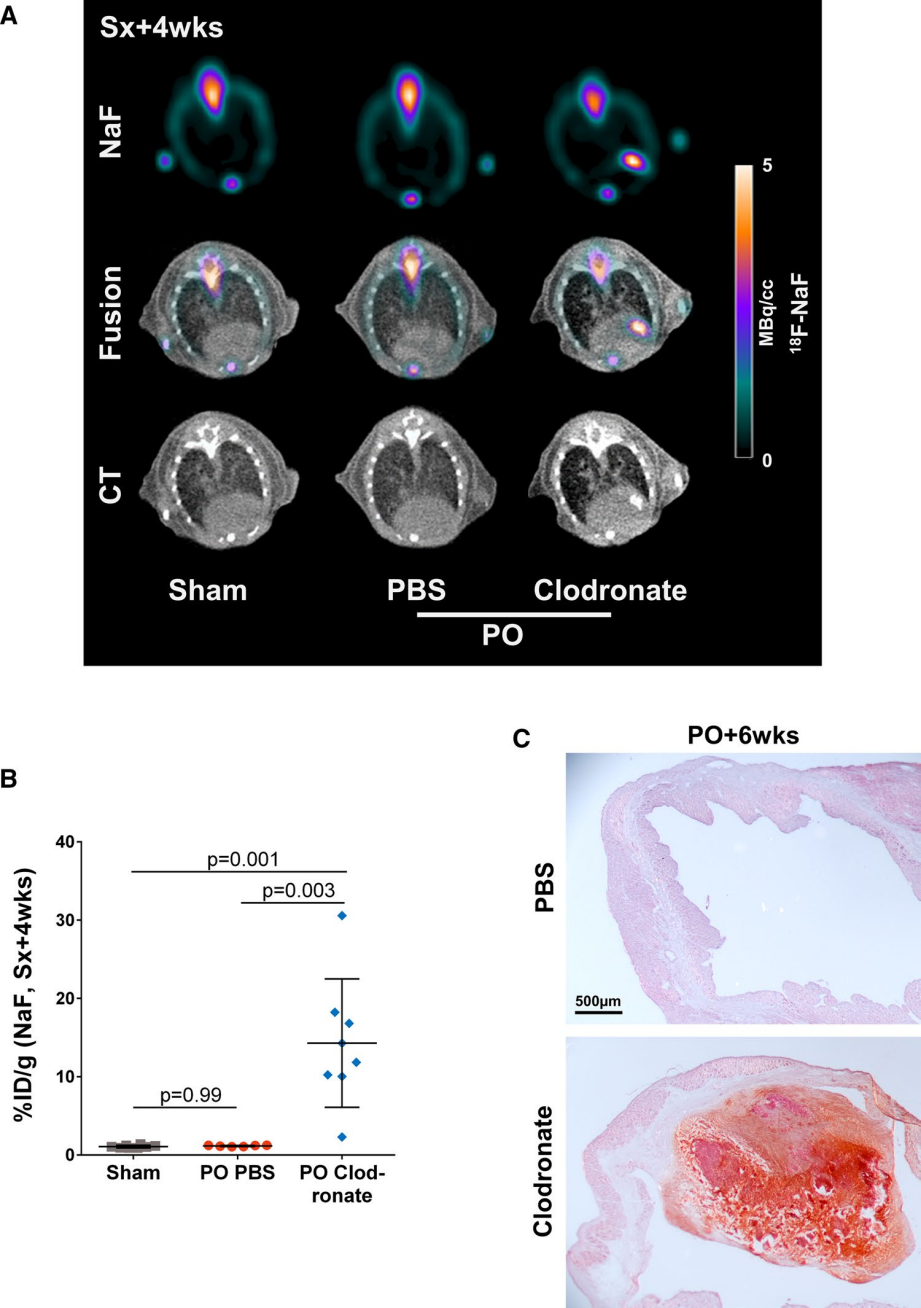
Calcification after macrophage depletion. **A** ^18^F-NaF PET images (colorscale) show active calcification in the thrombotic infarct region 4 weeks after permanent occlusion in macrophage depleted mice. A CT opaque corresponding area is visible in the co-localization CT. **B** Semi-quantitative image analysis demonstrates significantly increased ^18^F-NaF uptake in the infarct region in macrophage depleted mice compared to PBS-treated infarct mice and sham mice. **C** Alizarin red histology confirms calcification, mainly in the region of intraventricular thrombus at permanent occlusion MI+6 weeks. Statistics: one-way ANOVA with Bonferroni’s post-hoc test. *PO* permanent occlusion, *Sx* surgery, *wks* weeks

In aged infarcts, we observed a hyperintense signal localized to the perfusion defect in colocalization CT (Supplementary Fig. 2A). To evaluate the potential development of microcalcification, we performed ^18^F-NaF imaging at 4 weeks after permanent occlusion MI. While there was no appreciable cardiac uptake of ^18^F-NaF in PBS-treated infarct mice, nor in macrophage depleted sham mice, ^18^F-NaF was markedly elevated within the infarct region of macrophage depleted infarct mice, suggesting active microcalification (Fig. 4A, B). Quantitative analysis of CT revealed that the density of tissue calcification was significantly lower compared to bone at 4 weeks post-MI (Supplementary Fig. 2B). Alizarin red staining in cryosections at permanent occlusion MI+6 weeks confirmed the presence of calcification, which was mainly located in the thrombus region (Fig. 4C).

### The effects of macrophage depletion are similar in ischemia/reperfusion MI and permanent occlusion MI

**Fig. 5:**
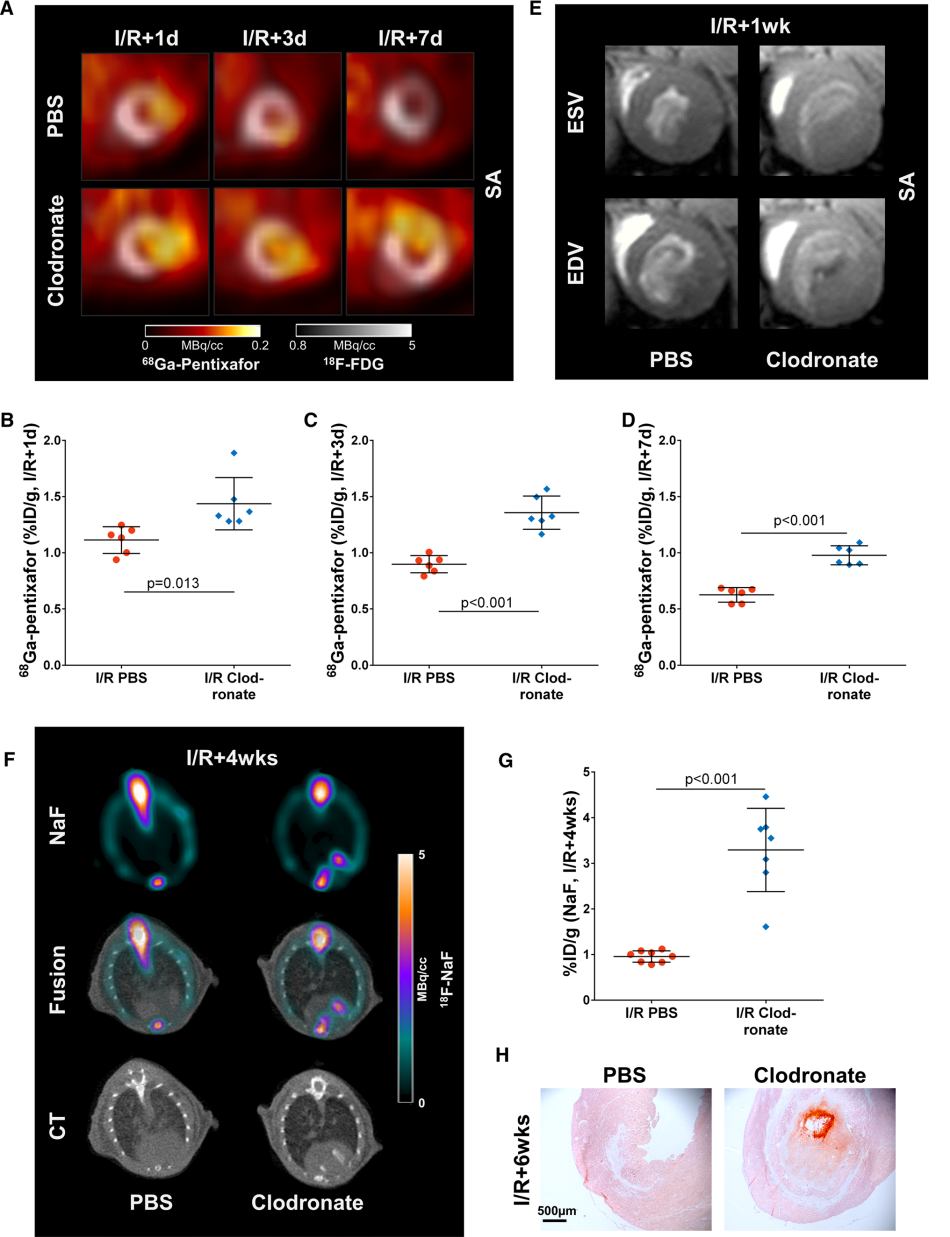
Macrophage depletion evokes similar changes after ischemia/reperfusion as in permanent occlusion. **A** Infarct CXCR4 expression in short axis cardiac PET images is significantly increased at **B** ischemia/reperfusion+1 day, **C** 3 days, and **D** 7 days after macrophage depletion compared to PBS. **E** Short axis cardiac magnetic resonance images reveal LV thrombus formation at ischemia/reperfusion+1 week after macrophage depletion, but not after PBS-treatment. **F** The thrombotic infarct area undergoes active calcification at ischemia/reperfusion+4 weeks after macrophage depletion, as demonstrated by ^18^F-NaF PET images. **G** The uptake of ^18^F-NaF is significantly increased in the LV after macrophage depletion compared to PBS. **H** Alizarin Red histology shows localization of calcification mainly in the LV thrombus. Statistics: Student’s unpaired *t* test with Welch’s correction where appropriate. *I/R* ischemia/reperfusion, *SA* short axis, *d* day, *wks* weeks

Chronic function and infarct size was not impaired by macrophage depletion prior to permanent occlusion MI (Fig. 1F, Supplementary Fig. 3), which we hypothesized may result from selection due to the increased incidence of acute ventricle rupture. Accordingly, we assessed imaging parameters in a model of ischemia/reperfusion MI, which typically results in non-transmural infarct localized to the inner layer of the myocardium without ventricle rupture [5]. Here, patterns of inflammatory response and thrombus development were similar to permanent occlusion, including significantly higher infarct CXCR4 expression at 1–7 days compared to PBS liposome-treated infarct mice, and distinct intraventricular LV thrombus formation (Fig. 5A–E, Supplemenatry Fig. 4). This confirms that thrombus formation after macrophage depletion does not require transmural infarction. Similar to permanent occlusion, at 4 weeks after ischemia/reperfusion, macrophage depleted infarct mice also exhibited increased uptake of ^18^F-NaF compared to PBS-treated infarct mice, indicating active calcification (Fig. 5F–H).

### Chronic functional impairment after macrophage depletion

**Fig. 6:**
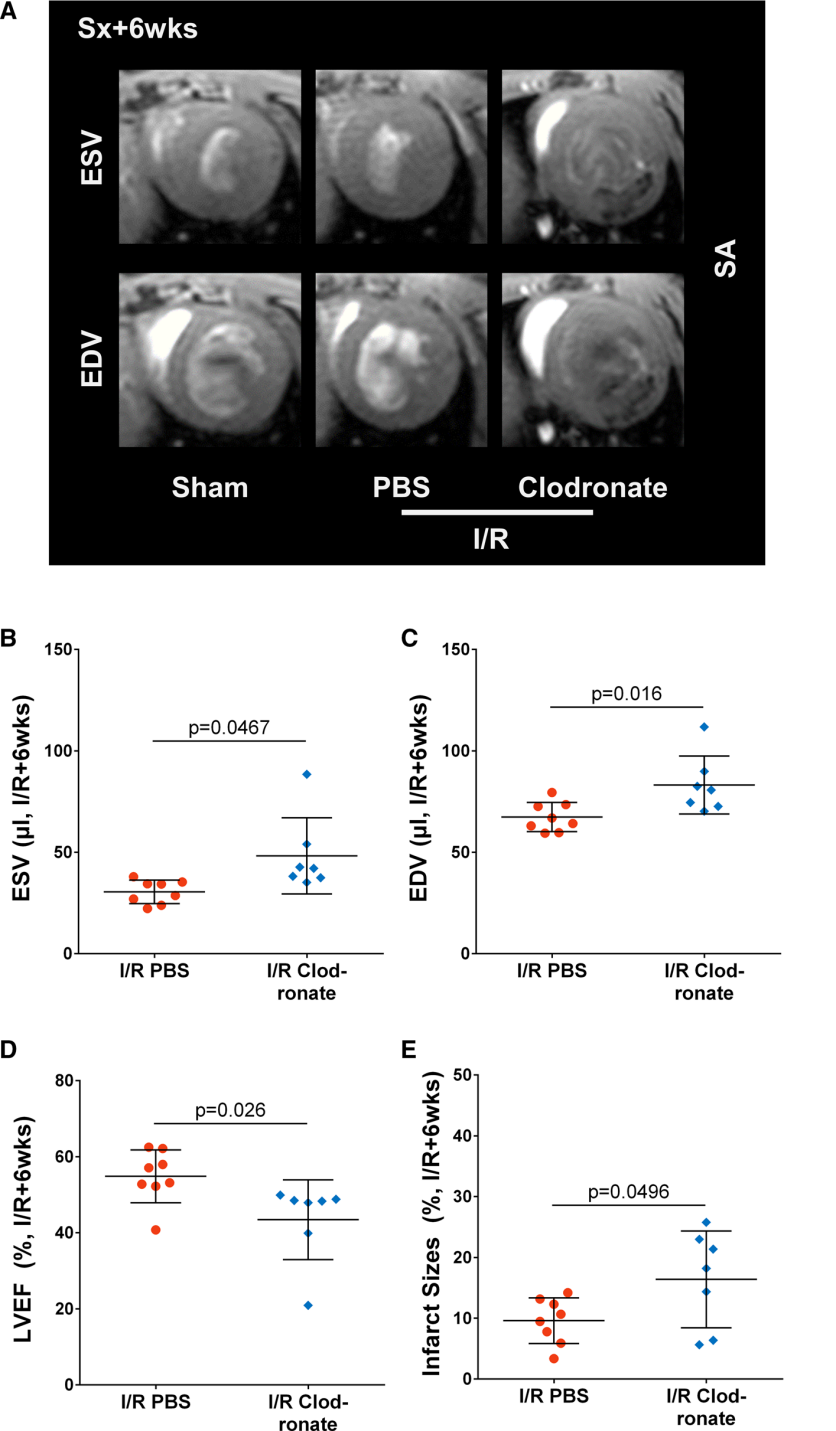
Macrophage depletion leads to adverse cardiac functional outcome after ischemia/reperfusion. **A** Cardiac magnetic resonance images at ischemia/reperfusion+6 weeks show pronounced LV dilatation in macrophage depleted infarct mice compared to PBS. **B** End systolic volume, and **C** end diastolic volume are significantly increased in macrophage depleted ischemia/reperfusion mice, leading to **D** significantly impaired LV ejection fraction. **E** The infarct size in macrophage depleted ischemia/reperfusion mice is significantly larger compared to PBS, suggesting increased infarct expansion. Statistics: Student’s unpaired *t* test with Welch’s correction where appropriate. *EDV* end diastolic volume, *ESV* end systolic volume, *I/R *ischemia/reperfusion, *LVEF* left ventricular ejection fraction, *SA* short axis, *Sx* surgery, *wks* weeks

No animals suffered LV rupture early after ischemia/reperfusion MI in the macrophage depleted or PBS-treated animals. However, at 6 weeks after ischemia/reperfusion, macrophage depleted mice showed significantly increased LV dilatation and lower LVEF compared to PBS-treated mice (Fig. 6A–D), suggesting impaired cardiac repair in the absence of peripheral macrophages at time of injury. Further, the final infarct size at 6 weeks post-ischemia/reperfusion was significantly larger after macrophage depletion compared to PBS, suggesting increased infarct expansion (Fig. 6E).

## Discussion

Infiltrating monocytes and macrophages critically contribute to cardiac healing and repair after ischemic injury. Complete peripheral macrophage suppression is detrimental to cardiac outcome. Using multiparametric multimodality imaging, we found that macrophage depletion by clodronate-loaded liposomes leads to impaired cardiac repair in two mouse models of MI with variable transmurality. Impaired repair features accumulation and persistence of granulocytes in the infarct territory, increased incidence of early LV rupture after transmural damage, and inferior late cardiac functional outcome. Notably, macrophage depletion also supports the formation of an intraventricular thrombus overlying the damaged infarct tissue. This intraventricular thrombus exhibits high levels of granulocytes and progressive calcification, which may contribute to further functional decline. This molecular imaging methodology bears implications for future strategies of image-based therapeutic guidance [13, 15].

We observed increased early mortality after permanent coronary occlusion in mice after clodronate depletion of macrophages. Consistent with this observation, macrophage depletion evokes increased ventricle rupture after cryoinjury-induced infarction [28] and higher incidence of embolism [10]. Timing of clodronate injection may influence the severity of impaired healing, as pre-surgery macrophage depletion, as in the present study, generated worse early outcome compared to later depletion from 2 days after injury [1]. Depletion of macrophages from 3 days after injury resulted in lower collagen deposition and angiogenesis [21]. These observations speak to the diverse roles of monocyte-derived macrophage subtypes in the healing process, wherein distinct subpopulations arise from the early pro-inflammatory phase immediately after ischemic injury to the later inflammatory resolution and repair phase some days later [23]. Since clodronate-loaded liposomes reduce circulating monocyte populations [25], the adverse outcomes observed in the present and other studies may reflect ineffective resolution of initial inflammation. TSPO-targeted PET delineated monocyte and macrophage infiltration, as depletion effectively ablated the imaging signal.

Despite the depletion of macrophages, the CXCR4 PET signal was persistently elevated over the first week after MI. This elevated signal corresponded to higher levels of Ly6G neutrophils both in the infarct region and prominently within a fixed intraventricular thrombus overlying the infarct damage. Prior studies have reported that macrophage inhibition leads to thrombus formation and embolism via secondary reduced secretion of factor XIIIa [10]. Notably, genetic deletion of factor XIIIa also leads to LV rupture, infarct expansion and LV dilation in mice after MI [20]. Alternatively, the absence of macrophages may lead to endothelial damage and dysfunction in the infarct region via persistent neutrophil and platelet activity as observed after laser-induced injury to generate fibrin and thrombus [4], and similar to our histologic observations after MI.

Macrophage depletion may impair inflammation resolution leading to worse chronic outcome, whereby the extent of the infarct after transient coronary artery occlusion expands in response to persistent inflammation. Since we used only a single dose of clodronate 24h prior to surgery and circulating monocytes and macrophages are repopulated after 5–7 days [25, 28], this observation may reflect delayed inflammation resolution leading to the larger infarct size and more severe remodeling among surviving animals.

Interestingly, we observed calcification associated with the infarct and overlying LV thrombus, visualized by ^18^F-NaF PET/CT. This observation may relate to osteocalcin expressed by abundant platelets at the injury site, which contributes to dystrophic calcification [8]. These mechanisms mirror clinical atherosclerosis, where microcalcification identified by ^18^F-NaF corresponds to macrophage-rich plaques imaged by ^18^F-FDG and other more specific agents [17]. The association between macrophage depletion, thrombus formation and calcification support a pathogenetic relationship between inflammatory cells and microcalcification. Such processes require further dedicated investigation.

We employed a multimodality molecular imaging approach in this study to enable longitudinal investigation of the transition from inflammation to healing in the infarct. Previous studies demonstrated that ^18^F-FDG accumulation in the infarct region was reduced after clodronate depletion [19], supporting specificity for inflammatory cells. We found that infarct TSPO expression is significantly decreased in macrophage depleted mice at MI+3 days and 7 days using ^18^F-GE180, whereas CXCR4 expression assessed by ^68^Ga-pentixafor was elevated compared to non-macrophage-depleted animals. These observations underscore the difference in the immune cell substrate of these compounds [2, 3, 13, 27]. This is consistent with observations in patients after MI, where prolonged CXCR4 signal elevation corresponds to worse outcome [13, 31]. Our study demonstrates the value of multi-tracer molecular *in vivo* imaging as a tool to investigate different targets in the same mouse longitudinally. The selection of imaging agent may be important to distinguish between monocyte/macrophage and granulocyte-rich inflammation, as persistence of either may have divergent outcomes.

## Limitations

There are some limitations to consider in this study. Owing to the already high complexity of our study design, we were not able to add further imaging sessions. Accordingly, we do not show the complete mechanism of thrombus formation and calcification after macrophage depletion. Even though defects in the endothelial layer of the endocardium likely initiate thrombus formation [10], the precise relationship between macrophages and endothelial cells needs to be elucidated by further mechanistic studies.

## Conclusions

In conclusion, macrophage depletion leads to impaired cardiac repair including increased accumulation of granulocytes, development of an intraventricular thrombus, and tissue calcification independent of infarct transmurality. Persistent granulocyte-driven inflammation may lead to infarct expansion, which results in higher incidence of LV rupture and more severe remodeling. Our work provides novel insights into the relevance of macrophages for adequate cardiac repair, and it may have implications for other cardiovascular diseases such as e.g. atherosclerosis. Importantly, our study also shows that multitracer molecular imaging enables investigation of longitudinal changes to biologic activity during cardiac repair, which may have implications for prediction of outcome and guidance of therapy in future translational projects.

## Supplementary Information

Below is the link to the electronic supplementary material.

Supplementary file1 (DOCX 872 KB)
