# Supplementary material for: Characterizing the transition from immune response to tissue repair after myocardial infarction by multiparametric imaging
Source: Basic Res Cardiol. 2022 Mar 11;117(1):14. doi: 10.1007/s00395-022-00922-x (PMC8917105; doi:10.1007/s00395-022-00922-x)
Supplement: Supplementary file 1 — Supplementary file1 (DOCX 872 KB) [file 395_2022_922_MOESM1_ESM.docx]

**Supplemental Information for**

**Characterizing the transition from immune response to tissue repair after myocardial infarction by multiparametric imaging**

Annika Hess, PhD, Tobias Borchert, PhD, Tobias L. Ross, PhD, Frank M. Bengel, MD,

James T. Thackeray, PhD

**Corresponding author**:

James T. Thackeray, PhD

Department of Nuclear Medicine, Hannover Medical School

Carl-Neuberg-Str. 1, 30625 Hannover, Germany

[Thackeray.James@mh-hannover.de](mailto:Thackeray.James@mh-hannover.de)

Tel. +49511 532 3358

Fax. +49511 532 3761

**This PDF file includes**:

Suppl. Table 1

Suppl. Fig. 1-4

Suppl. Video 1 caption

**Suppl. Table 1** Number of animals within the different treatment and surgery groups. Histo = Histology; d = day; AR = *ex vivo* autoradiography

|  | **Clodronate-Liposomes** | | | | **PBS-Liposomes** | | | |
| --- | --- | --- | --- | --- | --- | --- | --- | --- |
|  | **Imaging** | | **Tissue** | | **Imaging** | | **Tissue** | |
|  | **^18^F-GE180** | **^68^Ga-pentixafor** | **Histo** | **AR** | **^18^F-GE180** | **^68^Ga-pentixafor** | **Histo** | **AR** |
| **Sham** |  | **9** |  | **3d: 2** |  |  |  |  |
| **Permanent**  **Occlusion** | **18** | **19** | **1d: 3**  **3d: 5**  **7d: 4** | **3d: 3** | **11** | **12** | **1d: 2**  **3d: 3**  **7d: 2** | **3d: 1** |
| **Ischemia/**  **Reperfusion** |  | **7** |  | **3d: 2** |  | **8** |  | **3d: 1** |

**
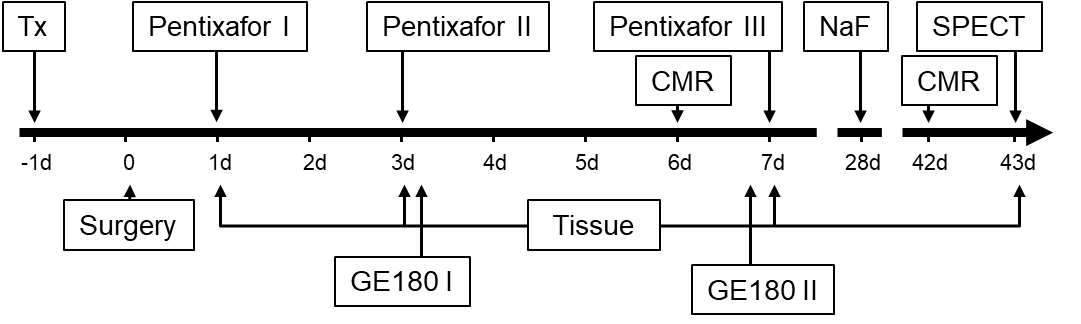
**

**Suppl. Fig. 1 Study design.** Tx = injection of clodronate-liposomes or PBS-liposomes, CMR = cardiac magnetic resonance, SPECT = single photon emission computed tomography, d = day


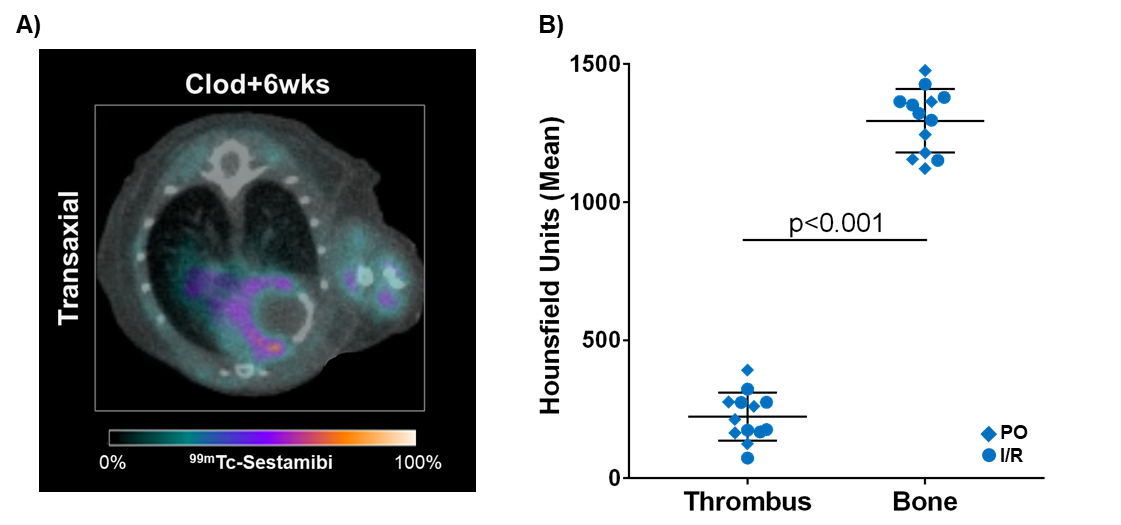


**Suppl. Fig. 2 Calcification of infarct area at permanent occlusion MI+6 weeks after macrophage depletion.** (**A**) Transaxial ^99m^Tc-sestamibi SPECT images and corresponding CT demonstrate calcification in the perfusion defect of the left ventricle at permanent occlusion MI+6 weeks after macrophage-depletion. (**B**) CT quantification 4 weeks post-infarct in macrophage depleted mice after permanent occlusion and ischemia reperfusion demonstrates a significantly lower tissue density in the calcified thrombus when compared to bone density. Statistics: Student’s paired t-test. Clod = Clodronate, PO = permanent occlusion, I/R = ischemia/reperfusion, wks = weeks

**
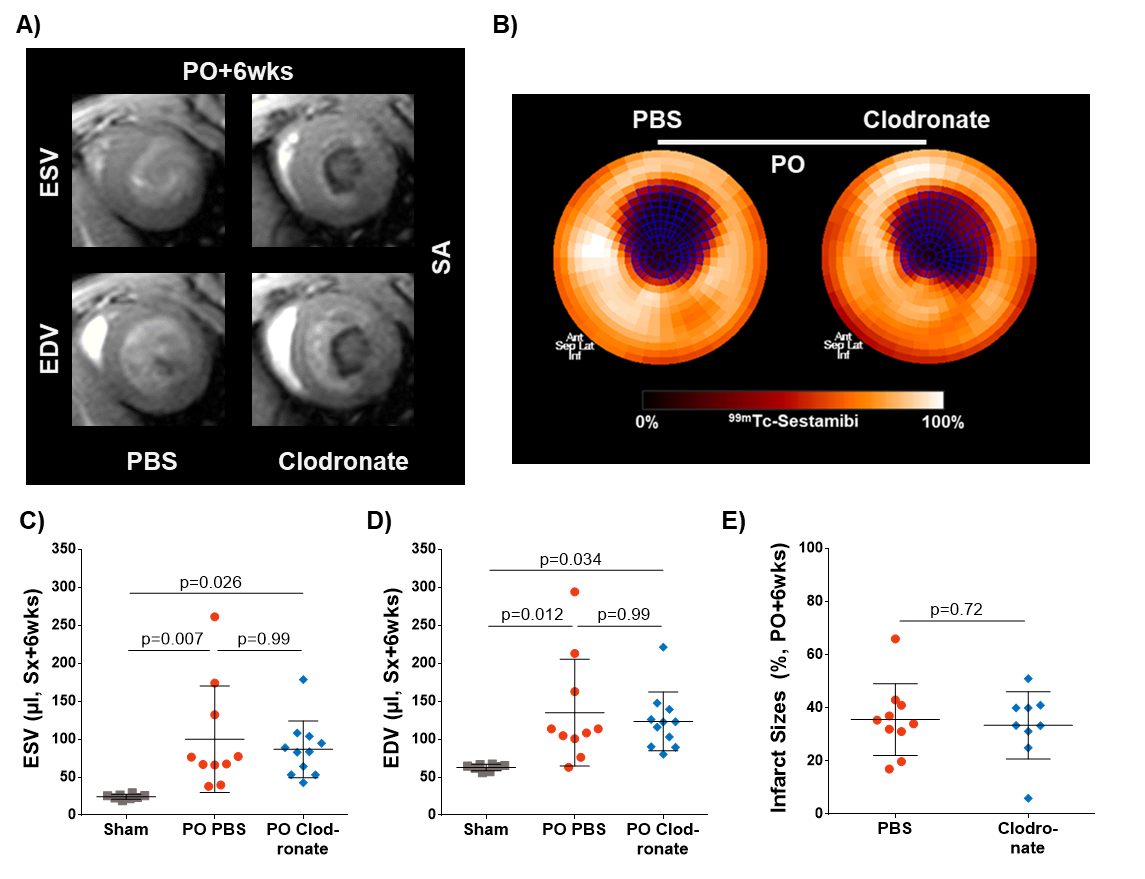
**

*****

*****

**Suppl. Fig. 3 Cardiac functional outcome in permanent occlusion MI mice**. (**A**) Representative cardiac short axis CMR images at 6 weeks after permanent occlusion demonstrate LV dilatation in both PBS-treated and macrophage depleted mice. (**B**) Representative perfusion SPECT polar maps in PBS-treated and macrophage depleted mice at 6 weeks after permanent occlusion. There is no significant difference between PBS-treated and macrophage depleted mice 6 weeks after permanent occlusion MI in (**C**) end systolic volume, (**D**) end diastolic volume, and (**E**) perfusion SPECT derived infarct size. Statistics: Student’s unpaired t-test, one-way ANOVA with Bonferroni’s post-hoc test. PO = permanent occlusion, ESV = end systolic volume, EDV = end diastolic volume, SA = short axis, Sx = surgery, * = intraventricular thrombus

**
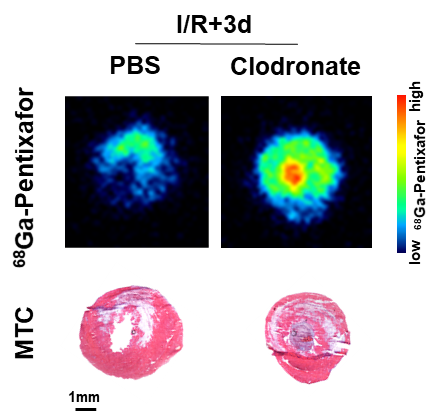
**

**Suppl. Fig. 4 *Ex vivo* autoradiography and Masson trichrome histology in ischemia/reperfusion MI mice.** *Ex vivo* autoradiography at 3 days after ischemia/reperfusion demonstrates increased uptake of ^68^Ga-pentixafor in the Masson trichrome derived infarct area, and intraventricular thrombus in macrophage depleted mice compared to PBS-treated mice.

**Suppl. Video 1** **LV thrombus is attached to infarct wall**. The intraventricular LV thrombus is attached to the infarcted myocardium at (**A**) permanent occlusion MI+1 week, and (**B**) permanent occlusion MI+6 weeks. LV = left ventricle, PO = permanent occlusion, wk(s) = week(s)
